# Conflicting perspectives on what constitutes fair compensation and benefits among research stakeholders in Malawi

**DOI:** 10.1186/s12910-025-01358-3

**Published:** 2025-12-23

**Authors:** Wezzie Nyapigoti, Kate Gooding, Blessings M. Kapumba, Wongani Mankhamba, Nicola Desmond, Deborah Nyirenda

**Affiliations:** 1 Malawi-Liverpool Wellcome Trust Clinical Research Programme, PO Box 30096, Chichiri, Blantyre 3, Malawi; 2 Liverpool School of Tropical Medicine, Pembroke Place, Liverpool, L35QA UK; 3 Oxford Policy Management, Ground Floor, 40-41 Park End Street, Oxford, OX1 1JD UK; 4 London School of Hygiene & Tropical Medicine, Keppel Street, London, WC1E 7HT UK

**Keywords:** Malawi, Compensation, Research benefits, Ethics, Qualitative research

## Abstract

**Background:**

International ethics guidelines such as the Council for International Organizations of Medical Science (CIOMS) recommend that research participants must be compensated for their time, travel, and inconveniences. However, there is continued debate on what constitutes fair compensation and benefits for research participants. We conducted a qualitative study and sought views of various research stakeholders on what they considered as appropriate compensation and benefits for study participation in Malawi.

**Methods:**

We employed a qualitative study design and conducted 10 focus group discussions (FGD) with frontline researchers, community leaders, research participants, study decliners and Community Advisory Group (CAG) members from medical research projects conducted in rural and urban Malawi. We also conducted 17 in-depth interviews (IDI) with researchers, ethics committee members and District Health Officers. Thematic and framework analysis was used to interpret the results.

**Results:**

Our findings showed that ethics review committee members, researchers and frontline researchers had a common understanding of compensation, informed by available literature. On the other hand, some community members understood that compensation was given due to harm resulting from study participation while others indicated that intended study benefits were not visible to the community. Our study participants also expressed concerns that offering unequal compensation based on study design, procedures and risks had the potential to make some individuals feel devalued if they received lower payment amounts compared to others.

**Conclusion:**

This study revealed conflicting perspectives on compensation and benefits among research stakeholders. While stakeholders involved in conducting research had a shared understanding of compensation as reimbursement for time and costs, some community members viewed it as redress for harm. The disconnect suggests unequal access to information about research ethics among research stakeholders. Varying compensation based on study procedures also raised concerns over perceived inequalities and feelings of being devalued. These results suggest a need for consensus-building through inclusive stakeholder engagement and open dialogue to co-design guidelines that balance individual reimbursement and community benefits to advance ethical research practices centered on respect for participants.

**Supplementary Information:**

The online version contains supplementary material available at 10.1186/s12910-025-01358-3.

## Background

Offering compensation to individuals who volunteer to participate in medical research is an accepted ethical research practice [1]. International ethics guidelines such as the Council for International Organizations of Medical Science (CIOMS), recommend that research participants have to be compensated for their time, travel costs, and inconveniences [2]. However, there has been ongoing debate on appropriate compensation to provide to research participants in medical research conducted in low-and-middle-income countries (LMICs) [3]. Some scholars have suggested that the amount of compensation should be commensurate with the extent of exposure to harm, be reasonable and based on local costs, so that it does not constitute undue inducement [1, 4]. While provision of research compensation is intended to counter exploitation of socio-economically disadvantaged groups, there are ethical concerns that providing compensation may unduly influence participation in research. For example, research in Malawi and other low-income settings has shown that some participants enroll in research to access compensation, treatment and other forms of reimbursements which might lead to exploitation [5]. On the other hand, there are ethical concerns around justice if studies provide low amounts of compensation in LMICs [5], and concerns that research may be conducted in such settings due to low costs [6]. Research ethics guidelines therefore need to carefully consider these factors to ensure that research participants are protected from exploitation and spared from unnecessary burden, physical, and emotional harms, while also demonstrating respect for their contributions and ensuring diverse recruitment without unduly influencing them to participate in medical research [6].

The issue of compensation and benefits in medical research conducted in LMICs presents complex ethical challenges [7]. According to CIOMS guidelines, benefits in biomedical research are potential health advantages (direct), non-health outcomes (indirect), or community/societal gains through research participation. Compensation on the other hand is defined as reimbursement of expenses, payment for time/inconvenience, and recognition for participation that should be fair but not unduly influenced. While research ethics guidelines state that any product developed from research should be available to participating research communities, some medical products may not benefit host communities in LMICs due to unaffordable costs [8]. Nevertheless, some scholars have argued that research projects must benefit host communities If they place significant burdens on public resources such as health facilities to ensure upkeep of public goods and minimize free riding, which refers to using community resources without adequately contributing to their maintenance or providing fair compensation to the host community [8]. There is, however, no clarity over what constitutes meaningful benefit or which groups of people are entitled to those benefits [8]. Inconsistencies on what constitutes fair benefits are also reflected in international guidelines on benefit sharing [9]. For instance, Lairumbi and colleagues (2012) reviewed 16 guidelines (seven international and nine country-specific) on benefit sharing in resource-poor settings and reported that the main debates on benefit sharing relate to who is receiving the benefits, benefits to the wider community in which research is located and the societal benefits that are associated with successful completion of research [10].

These ongoing debates and concerns mean that further work is needed to determine appropriate benefits and compensation when working with socio-economically disadvantaged communities. There is currently limited empirical research on appropriate levels of compensation or benefits for the community as a whole, or how these might vary between different types of research [11]. In addition, there are gaps in the literature on what constitutes fair individual or community benefits. Household-level Randomized Controlled Trials (RCTs) exemplify this ethical dilemma, where randomized participants receive direct benefits while neighboring households bear potential burdens without corresponding compensation. This highlights limitations in current research compensation frameworks, emphasizing the need to develop approaches that meaningfully account for the complex dynamics of collective and individual research participation [12, 13]. Furthermore, while communities should be consulted to enhance protection from exploitation, decisions on appropriate study benefits and fair compensation are usually pre-defined by researchers, Research Ethics Review Committees (RECs) and other research stakeholders, without consulting communities or considering participants’ views.

The National Commission of Science and Technology, which oversees guidance on ethical conduct of research in Malawi, recommended in 2018 that all research participants should receive $10 per study visit. This however prompted debate among the research community about whether this was ethical in the Malawian setting or whether the $10 amount would unduly influence research participation given that more than 75% of the population live on less than $1.25 a day. In Malawi, previous research has shown that research participants enroll in research to access compensation and benefits, rather than for altruistic reasons [14]. In addition, Mfutso Bengo and colleagues (2008) found that some participants decline to participate in research that does not provide any individual benefit [15]. There were also concerns among research stakeholders that the level of compensation should vary depending on study design, duration of study and the level of burden for participants, and that the $10 might not be feasible for all research projects, particularly students with no research funding. Discussions among the researchers and regulators led to the development of revised and more detailed guidance to ensure transparent and fair remuneration of research participants in Malawi [16]. However, this guidance was developed without seeking input from research communities or incorporating their perspectives on participation incentives and compensation. This study was therefore undertaken to explore the views of research stakeholders, including community members on compensation and benefits for research participants, to help inform decisions on appropriate guidance.

## Methods

A qualitative study design was used to answer the research question to allow us to explore and describe research stakeholders’ understanding and perspectives of research compensation and benefits. We used Focus Group Discussions (FGDs) and In-Depth Interviews (IDIs) to explore the views of research stakeholders on appropriate levels of compensation and benefits in Malawi. The topic guides used for both IDIs and FGDs were developed specifically for this study to explore stakeholder perspectives on appropriate compensation and benefits for research participants in Malawi. These guides contained open-ended questions designed to elicit views on what constitutes fair compensation and benefits.

We conducted 17 IDIs with researchers [5], REC members [5], researchers from various research institutions [5] and district health officers [2]. Ten FGDs were also conducted with study participants [2], people who had declined to participate in a research study [2], Community Advisory Group (CAG) members [2], community leaders [2] and field workers/nurses [2] (see Table 1). In this paper, all individuals who participated in the research and consented to take part in FGDs will be referred to as ‘study participants’ while the term ‘research participants’ will refer more broadly to anyone who takes part in research. The term ‘research stakeholders’ will be used to describe various participant groups involved in IDIs, including CAG members, REC members, community leaders, researchers, and field workers.

**Table 1:** Stakeholders involved in FGDs and idis

Data collection method	Stakeholders	Number of participants	
		Male	Female
Focus group discussion	MLW field workers (1 FGD)	6	6
	MLW nurses (1 FGD)	4	4
	Coummunity Leaders urban (1 FGD)	5	4
	Coummunity Leaders Rural (1 FGD)	4	4
	MLW study participants urban (1 FGD)	5	5
	MLW study participants urban (1 FGD)	5	4
	MLW study decliners urban (1 FGD)	4	5
	MLW study decliners rural (1 FGD)	4	4
	MLW Community Advisory Group members urban (1 FGD)	4	5
	MLW Community Advisory Group members rural (1 FGD)	5	4
In-depth interviews	MLW Researchers	3	2
	Researchers from other research institutions	3	2
	Ethics committee members	3	2
	District Health officers	2	-
Total		108 Participants	

Interview locations were selected to consider different socioeconomic conditions in urban and rural Malawi. The study was conducted in Blantyre which is the biggest commercial city in Malawi [17] and Chikwawa, a rural field site. About 53% of Malawi’s poor population live in the Southern region, and of these about 89% live in rural areas and 11% live in urban areas. To compare perspectives between rural and urban areas, and between lower and middle-class neighborhoods, IDIs and FGDs were conducted in Ndirande, Blantyre district (urban) and Chikhambi village, Chikwawa district (rural). Ndirande is classified as a middle and poorer-class township, within Blantyre while Chikhambi village is classified as rural. The selected sites fall within the catchment area of health centers where Malawi Liverpool Wellcome Programme (MLW) carries out research projects.

### Participant recruitment

To make sure that participants were well informed about the study and to enable them to make an informed decision, the following procedures were followed for both FGDs and IDIs.

### Focus group discussions

We worked with Principal Investigators from selected studies and other relevant authorities to seek permission to contact study participants and study decliners. We also engaged the Clinical Research Support Unit (CRSU) and Science Communication department at MLW to seek permission to contact frontline researchers, community leaders, and CAG members. Thereafter, we approached all the participants through phone calls, emails or in person to introduce them to the study. Participants who showed willingness through written informed consent were booked and invited to FGDs. Before each FGD, we shared the study information leaflets and explained the study details. Participants were given enough time after going through the study information leaflet to ask questions and seek clarification on any issues they did not understand. All FGD participants gave their written or oral informed consent and an impartial witness recommended by illiterate participants was involved in the consenting process. All FGDs took place at a venue selected by participants themselves.

### In-depth interviews

We worked with CRSU at MLW to get contact details of REC members, researchers, and DHO staff. All the potential participants were approached via phone calls or emails to book appointments. Participants who accepted our invitation were given time to read the study information, ask questions and seek clarification where relevant. We conducted interviews at a venue convenient and secure to safeguard confidentiality. Written informed consent was sought before each interview.

### Data collection and analysis

For both IDIs and FGDs, we developed a topic guide with open-ended questions on stakeholder views regarding appropriate compensation and benefits for study participation. All the IDIs and FGDs were facilitated and recorded by WN. Thereafter, all the audio recordings were transcribed and translated from Chichewa to English by WN, WM and checked by DN. After going through the first transcripts from the FGDs, IDIs and field notes, WN developed codes emerging from the data, in discussion with DN, BK and KG. A coding framework was developed in QSR NVivo 11 where transcripts were imported. The analysis employed both thematic analysis to identify descriptive and interpretive themes and framework analysis to enable systematic comparison of perspectives between different stakeholder groups (participants, decliners, researchers, REC members, community leaders). Data analysis was conducted iteratively, allowing emerging issues to inform subsequent data collection, while findings were organized around key themes such as: perspectives on research compensation, perspectives on research compensation based on study procedures, and views on individual versus collective benefits. The presentation of findings strategically used direct quotes from participants to illustrate themes, with quotes carefully selected to represent different stakeholder perspectives and geographic locations (urban vs. rural), and each quote was attributed with participant type and focus group/interview identifier.

### Ethics approval

This study was conducted in accordance with the principles of the Declaration of Helsinki. Ethics approval was obtained from the Kamuzu University of Health Sciences (KUHES) ethics committee (formerly known as University of Malawi, College of Medicine Research Ethics Committee P.06/18/2420) and the Liverpool School of Tropical Medicine in the United Kingdom (18–057). All participants provided informed consent prior to participation. For illiterate participants, the consent process was approved by the ethics committee. Oral informed consent was obtained in the presence of an impartial witness chosen by the participant. The witness read the study information leaflet to the participants, ensuring they fully understood the study procedures, potential risks, and their rights. Only after demonstrating comprehension and voluntarily agreeing to participate was informed consent documented. Additional approvals were obtained from research institution directors, District Health Officers, Principal Investigators, and study coordinators before commencing data collection.

## Results

The study findings revealed diverse perspectives regarding appropriate compensation and benefits for research participation in Malawi and suggestions for research compensation. Our analysis also showed that research stakeholders perspectives varied based on their social position, research ethics literacy, and research experiences. These results highlight complexities in responding to individual and community preferences, gaps in understanding of research ethics particularly among community members, and opportunities for building greater consensus through improved communication, consultation, and transparency. This section presents the key themes in detail, elucidating the nuances within stakeholder perspectives on this ethically complex terrain.

### Perspectives on research compensation

The results show that perspectives of study compensation varied among research stakeholders. Most of the study participants from both urban and rural settings understood compensation as payment given to study participants, whether material/money or in kind to compensate for time and transport costs. These participants had enrolled in research, and they heard about the rationale for compensation from frontline researchers.Other participants also explained that compensation was given to minimize burdens through invasive procedures and the pain they underwent in fulfilling study procedures as shown in the quote below.

*“It (Compensation) is something given to us participants for unpleasant situation or if we have been harmed whether knowingly or unknowingly” (Study participants CK FGD 3)*

Others perceived compensation as a way of respecting study participants for taking time to share information and samples valuable to research. Compensation was therefore seen as one way of appreciating and showing respect for the study participant’s contribution.

In contrast, few participants from rural settings stated that compensation was given to incentivize study participation and to increase study enrollment. Such participants reported that compensation was given to them due to their low socio-economic status to influence them to participate in research as shown in the following quote.

*“Compensation is something which has been prepared consciously considering our status (poverty). As for me*,* I can accept and appreciate it” (study decliner CK FGD 05)*

Some frontline workers and research staff also had similar views and reported that compensation was necessary in their professional duties to incentivize study participation and increase study enrollment. Offering compensation was viewed as useful, particularly in studies with follow-up visits to encourage participants to fulfill the required study procedures and ensure successful completion of the study.

*“… Compensation is necessary to ensure that participants come*,* we give them transport*,* as a motivation so that they may come again to fulfill remaining study visits” (Male frontline worker CK FGD O1)*

*“Compensation is a financial assistance which helps participants to fulfil study visits if the study has got follow-ups” (MLW Researcher IDI 12)*

On the other hand, some community leaders thought that compensation was given to study participants because of harm induced through research participation.This was probably due to confusion with workers’ compensation for accidents or the vernacular translation of compensation ‘*chipepeso*’ which means ‘to apologize or ask for forgiveness’. This harm was, however, seen as intentional, with the researcher’s full awareness that the study outcome will cause harm to some participants.

*“The word compensation to me means someone has done something which is not good and then he says sorry for the wrong I have done for you*,* then you (researchers) can give him/her something” (Female Comm leader BT FGD 10)*

Our findings also show diverse perspectives on what is considered appropriate compensation for research participation. Some study participants from the urban setting explained that they refused to take part in some research projects due to low or unacceptable compensation. This view was commonly held among urban participants, and they reported that materials such as soap, sugar, bed nets, basins and study-branded T-shirts, which were being given to study participants as tokens of appreciation, were inadequate. Such participants felt that these goods could not be used to pay for transport fares and other out-of-pocket costs, and therefore did not adequately compensate for study participation as explained by a study decliner below.

*“I once declined to join a study…… because they told me that they will give me a study branded T-shirt at the end of the study…. How can I pay my bicycle fare with a T-shirt*,* what about my time spent in this study…. then I told them I can’t join your study*,* look for someone elsewhere” (study decliner CK FGD 05)*

Similarly, some REC members stated that there was no adequate compensation for time spent on studies and inconveniences incurred during study participation. They stated that what was referred to as a study compensation was transport reimbursement to fulfil study visits as explained by a female REC member below.

*“You see most studies here in Malawi only provide transport reimbursement…which is leading to under-compensating our participants” (Female REC member IDI 13)*

On the other hand, some research staff felt that providing medical care during research participation was more appropriate compensation than offering material benefits. This was a commonly held view among researchers conducting clinical trials who reported that medical care provided to study participants during study participation was adequate compensation, unlike monetary compensation. They felt that the presence of highly trained staff, availability of medical supplies, consultation rooms and adequate wards to admit patients and offer quality services lacking in government hospitals was justifiable compensation as shown below.

*“In my study I provide better care to participants*,* put them in nice wards*,* they meet well-trained clinicians and receive good medicine which is a more justifiable benefit than monetary or material compensation” (researcher*,* IDI 04)*

### Perspectives on research compensation based on study procedures

Most participants in urban and rural settings reported that providing compensation based on study design and procedures could potentially lead to exploitation. They explained that basing compensation on assessments of risks or burdens is subject to the researcher’s subjectivity as to whether the study procedures warrant high or low levels of compensation.Since frontline workers are often alone in communities without supervision, they can easily exploit communities and give low compensation. Offering variable compensation in the same setting could also make some participants feel less important if they received lower amounts compared to others. This could affect data quality and recruitment, if participants joined studies that offer better compensation as explained by one FGD participant below.

*I think providing compensation*,* based on study design and procedure can lead to under-compensation as well as lead to many refusals*,* because if we are compensated less*,* we can feel that we are not important to the study (Study participant CK FGD03)*

Similarly, some REC members and researchers argued that allowing researchers to set compensation presents a conflict of interest. They stated that an independent body should determine appropriate compensation, to minimize exploitation. Others also said that all participants deserve equal compensation for contributing to scientific knowledge production as shown below.

*“My take is that the objective for study participation is the same. Compensate them appropriately regardless of the study design” (Researcher from other institutions IDI 05)*

*“When we start paying our participants based on risks it will depend on how one weighs the risk in his/her study*,* the one to decide this is not supposed to be the one designing the study*,* for this will lead to bias.” (REC member IDI 17)*

Furthermore, some researchers, CAG members, and study participants stated that additional factors such as transport costs, procedural burdens and risks should help determine additional compensation costs. They suggested a standardized base rate, with additional reimbursement or materials provided as tokens of appreciation.Other researchers said stakeholders at the study settings should offer guidance on research compensation and determine appropriate compensation for that context. The different perspectives among research stakeholders were influenced by their roles, priorities, and perspectives of the research process. Study participants often prioritized practical concerns, such as transport costs, procedural burdens, and risks, shaped by their lived experiences.

### Perspectives on study benefits

Our findings also show varied perspectives among research stakeholders regarding study benefits. Responses from some research stakeholders, however, demonstrated some confusion between study benefits and compensation. For instance, the majority of community stakeholders from the rural setting including community leaders, study participants, and CAG members stated that monetary and material resources, diagnosis and medical treatment were all study benefits. They also mentioned that other benefits for study participants in clinical trials included short waiting times to see a clinician as shown below.

*“Our people are benefiting a lot when they participate in a study. They go through several (laboratory) tests*,* their problem is known and immediately they start treatment. Their problems are solved. They get T-Shirts*,* bed nets*,* basins*,* pails*,* and pots*,* they benefit a lot from these studies” (Male Community leader CK FGD 09)*

*“Money given to us after participating in the study are what we call benefit from research” (Decliner CK FGDs 05)*

On the other hand, participants from the urban setting thought study benefits were outcomes and interventions which came after study implementation even though these were not visible to them. The differing perspectives were shaped by the broader structural context. In rural settings, the presence of poorly resourced government facilities made clinical services offered by researchers more appealing, whereas in urban settings, where services were relatively better resourced, such improvements were less appealing.

*“We are always told that we will benefit from the outcome of the studies*,* but it’s hard to see the research benefits” (Female*,* MLW research participants BT FGDs 04)*

Similarly, some REC members, researchers, and DHOs believed that research should benefit the broader community or society rather than providing direct benefits to individual participants, making these benefits less visible to participants.

They also mentioned that medical care offered to study participants was not a benefit, but the study procedure to answer the research questions.

***“****In my explanation I have said that there are no individual benefits in research*,* if there are such cases*,* that is wrong (…) The benefit in most case is for the community*,* like College of Medicine has been working in Lungwena and constructed a Health center*,*” (Male REC member IDI 15)*

*“The way I see (medical) care is not a study benefit*,* because you are providing these things as a means to achieve the scientific rigor that you need*,* and most people who are participating in the research do not directly benefit from research*,* it (research benefits) will come to their community later on” (Female DHO IDI 02)*

A contrasting view of benefits was held by participants who had declined to take part in research studies. They explained that the outcome of study benefits was not visible to communities. This was probably because the study results took time to be translated and implemented. In addition, they stated that some studies failed to achieve the research objectives to improve health.

### Suggestions on study compensation

Research stakeholders such as community leaders, study decliners, researchers, and REC members suggested that greater consultation was needed when determining appropriate compensation. They explained that community members were best placed to articulate their own preferences on compensation, rather than outsiders. Responses from some community stakeholders also demonstrated that they felt research relationships were unequal, and their voices were excluded in decision-making. Involving communities could make participants feel valued as explained by one leader:

*“There is need to engage community leaders because we live together with people who participate in studies here in our villages*,* we hear their concerns*,* and what they need as compensation” (Male community leader CK FGD 09)*

Some research stakeholders suggested removing compensation details from consent forms, to avoid unduly influencing research participation while others recommended standardized pay, to avoid suspicions of embezzlement of money by frontline workers when compensation varies. On the other hand, some researchers felt research budgets should determine appropriate compensation, with well-funded studies compensating better as indicated below.

*“The first thing we look into is money*,* what is the budget in this particular study*,* sometimes you find a researcher has got enough money in his/her budget*,* the salary is huge plus other allowances*,* but they are saying I can’t compensate because I don’t have money” (Female REC member IDI 13)*

## Discussion

The findings highlight that research stakeholders views on compensation and benefits in research were strongly influenced by their specific roles. Those directly involved in the studies, such as study participants or those who declined participation, tended to focus on individual compensation shaped by personal needs. In contrast, oversight bodies like REC members emphasized fair individual compensation and community benefits. Similarly, frontline workers and researchers valued both individual and public health benefits, while community leaders and advisory group members leaned more toward community-level benefits. Overall, perspectives aligned with the stakeholders’ level of involvement, with those closer to the research process prioritizing individual benefits and those more removed emphasizing broader community benefits.

More generally, there was a lack of consensus on appropriate compensation and benefits, with some stakeholders suggesting medical care offered during research participation as adequate and others suggesting standardized monetary compensation. Stakeholders’ views also differed on whether compensation should be standardized or vary based on duration of study and other out-of-pocket expenses. Additionally, there were conflicting perspectives of research benefits, to either benefit the individual participant or the broader community. Transparent communication of study compensation and research benefits through ongoing community meetings, can help to improve community understanding of research benefits and compensation and minimize community unmet expectations from research participation [18]. We argue that determining appropriate compensation to research participants requires collaborative efforts between researchers and community members to improve mutual understanding of research compensation and benefits and build trust between the researchers and participating communities. In addition, there is need for more clear generic research communication on the distinction between study compensation and research benefits [18]. We also support the idea of Harvey and colleagues (1992) to openly inform participating research communities about research compensation arrangements [19].

Our findings demonstrated that excluding community stakeholders from compensation discussions has contributed to divergent interpretations of research compensation, with participants understanding it primarily as recompense for harm or injury. This is consistent with both common dictionary definitions and standard clinical trial consent language that addresses compensation for injury. This interpretation differs from researchers’ intended meaning of compensation as participation remuneration. The complexity is compounded with the local language translation of compensation (*chipepeso*) which means ‘to apologize or seek forgiveness’ thereby reinforcing the harm related interpretation. Rather than viewing this as a simple misunderstanding, these findings highlight the need for researchers to recognize the multiple legitimate meanings of compensation and work collaboratively with communities to establish shared terminology. This requires moving beyond one way explanation during community meetings and consent processes towards genuine dialogue that acknowledges different but valid interpretations. Communities must therefore be engaged to identify other appropriate vernacular phrases to communicate compensation and minimize misunderstandings of research compensation as payment for research-induced harm.

Challenges to translate local biomedical or research ethics concepts from English into local languages have been widely reported in LMICs [20, 21]. These challenges to communicate research procedures or research ethics in locally relevant terminologies can result in misunderstandings and detrimental rumors which can affect research participation in LMICs. For instance, a qualitative study conducted in South Africa reported that some research participants perceived research involving biobanking as bad research because it involved storing human blood in a fridge [22]. Similarly, compensating research participants for drawing blood samples has been interpreted as buying blood for evil purposes in most African countries [23]. Some scholars have argued that such misinterpretations should not be dismissed as ignorance or superstition, researchers must however engage with these misunderstandings to improve community trust in research [24]. Addressing such misunderstandings can minimize further marginalization of disadvantaged communities who do not participate in research or adopt effective interventions that can improve their health [25].

Our findings further highlighted a disconnect between research benefits as discussed in international ethics guidelines and how research participants perceived them. These results are in line with previous research conducted in Malawi that some research participants fail to differentiate between humanitarian aid and medical research, hence they find research benefits to be invisible as compared to humanitarian aid which directly benefits them [26]. While existing ethics guidelines focus on collective benefits to the society [2], our findings show that research participants preferred individual benefits such as medical care while non-research participants preferred collective benefits. To minimize confusion between research benefits and humanitarian aid, the distinction between the two must be made clear during community meetings.

Most of the international ethics guidelines recommend that research participants must be compensated for their time and expenses [18], however decisions on appropriate compensation are often determined by researchers and exclude research participants [5, 14]. Our findings show that the majority of study participants preferred standardized amounts of compensation rather than varying compensation based on risks, which is in line with what others have proposed [27]. Considering the four models of research payments to incentivize, compensate for time and contribution, reimburse for actual costs and offer token of appreciation [1], these results show that study participants preferred the standardized wage payment model which compensates for time and contribution. These results therefore show that existing ethics guidelines could be relevant to the socio-economic context through community consultation [8]. The varied participants’ perspectives however underscore the complexity inherent in determining fair compensation for research participants or reaching consensus during public consultations [28]. Nevertheless, the diverse views reflect that communities are not homogenous and their diverse views need to be taken into consideration to improve compensation practices [14]. Despite the complexity to respond to diverse views, these findings suggest a need for inclusive deliberative processes that value different viewpoints to develop locally relevant, ethically sound compensation guidelines [8].

## Conclusion

This paper reports on the nuanced complexities inherent in determining appropriate compensation and benefits for research participants in resource-limited settings. While there is disagreement on suitable compensation, we highlight the need for community consultation in developing locally relevant, participant-centered compensation policies. Core principles endorsed by stakeholders are fair reimbursement for time and costs, avoiding undue influence, and standardized pay schemes to prevent inequities. Perspectives diverged based on proximity to research, but all agreed compensation should uphold dignity and prevent exploitation. These results affirm compensation as an ethical obligation that must be shaped through inclusive deliberation to balance individual benefit and community benefit. A consensus workshop involving all relevant stakeholders and ongoing dialogue with the community is necessary to develop context relevant guidelines.

## Supplementary Information

Supplementary Material 1.

## Data Availability

The datasets generated and analyzed during the study are not publicly available due to the sensitive nature of the qualitative data and to protect participant confidentiality, but de-identified data are available from the corresponding author upon reasonable request and subject to ethical approval from the relevant ethics committees.

## Abbreviations

CAG: Community Advisory Group
CRSU: Clinical Research Support Unit
CIOMs: Council for International Organizations of Medical Science
DHO’s: District Health Officers
FGD: Focus Group discussion
IDI: In-depth interviews
KUHES: Kamuzu University of Health Sciences ethics committee
LMIC: Low- and medium-income countries
MLW: Malawi-Liverpool Wellcome Trust Clinical Research Programme
REC: Research Ethics Committee
RCTs: Randomized Controlled Trials
