# Supplementary material for: Conflicting perspectives on what constitutes fair compensation and benefits among research stakeholders in Malawi
Source: BMC Med Ethics. 2025 Dec 23;27:9. doi: 10.1186/s12910-025-01358-3 (PMC12836829; doi:10.1186/s12910-025-01358-3)
Supplement: Supplementary file 1 — Supplementary Material 1. [file 12910_2025_1358_MOESM1_ESM.docx]

**Appendix 1: Topic guides for REC**

All topic guides will be used flexibly and adapted during the research in response to pilots and emerging findings

**Research ethics committee member interview guide**

**Views of research stakeholders about appropriate benefits and compensation for study participation**

**Roles in COMREC/NHSRC**

What are your roles in the committee? How long have you been involved in the committee?

**Views on compensation and benefits**

What do you understand about compensation and benefits?

Have you seen any proposals where compensation and benefits will be provided?

How are decisions made on appropriate level of compensation and benefits on study participation?

What factors are considered important when making decision on appropriate level of compensation and benefits on study participation?

What do you think is fair compensation and benefits on study participation and why?

What do people think should be considered when deciding on levels of compensation and benefits?

Do people feel that compensation should depend on study type or what participants have to do? E.g. whether samples are taken, number of visits, transport visits, transport distance, international trial level of risk and side effects. If so, how does this affect compensation?

**Suggestions for future appropriate level of compensation and benefits.**

How people would rate the burden, discomfort of different procedure or what they think about calculating compensation based on reimbursement, compensation and incentives?

What are the steps that should be followed when considering appropriate levels on compensation and benefits on study participation?

Would the necessary steps be the same if the study design is different? E.g. the burden of the research.

How should the process vary depending on different study design?

Do they have to submit ethics applications specifically for appropriate levels of compensation and benefits on study participation?

THANK YOU.

**Appendix 2: Topic guide for DHOs/PIs**

**DHOs/PIs interview guide**

**Views of research stakeholders about appropriate benefits and compensation for study participation**

**Experience of research**

Please tell me about your research experience and the kinds of studies you are involved in.

**Views on compensation and benefits on study participation**

Have you been involved in any study where compensation and benefits has been provided to participants?

What kind of compensation or benefit was shared?

What processes were used for this?

What was the purpose of providing these compensation or benefits?

What did you see as benefits of providing compensation or benefits on study participation?

Did you feel there were any risks?

Is there anything you would change about the process now or do differently?

If compensation and benefits was not provided:

Why was this decision made?

Were there any disadvantages of not providing compensation or benefits?

What process, if any, would have made providing compensation or benefits possible?

**Recommendations or suggestions for future data sharing**

How people would rate the burden, discomfort of different procedure or what they think about calculating compensation based on reimbursement, compensation and incentives?

What are the steps that should be followed when considering appropriate levels on compensation and benefits on study participation?

Would the necessary steps be the same if the study design is different? E.g. the burden of the research.

How should the process vary depending on different study design?

Do researchers have to submit ethics applications specifically for appropriate levels of compensation and benefits on study participation?

THANK YOU

**Appendix 3: Topic guide for community representatives, community leaders and field workers/nurses (English version)**

**Community research representatives, community leaders and Fieldworkers/nurses – focus group guide**

**Views of research stakeholders about appropriate benefits and compensation for study participation**

**Community and staff experience of research**

*Fieldworkers/research nurses:*

How long have you been working/ involved with MLW studies?

What studies have you been involved in?

What were the studies about?

*CAG members and community leaders:*

What research projects have been conducted in your community / do you have experience of?

Have you had any experiences of studies providing compensation or benefits?

**Views on appropriate levels of compensation and benefits on study participation**

Were these studies providing compensation or benefits to participants?

If compensation or benefits was provided:

What kind of compensation or benefit was shared?

How did you feel about providing these compensation or benefits to participants?

What do you think is fair compensation and why?

What do you think should be considered when deciding on compensation levels?

Do you feel compensation depends on study type or what participants have to do? If so how?

What reactions do you hear from communities regarding compensation and benefits offered by studies? How does this vary?

**Vignettes**

Vignettes will be presented to participants indicating scenarios with different types of research (e.g. clinical trial, community-based study, ill or healthy volunteers) to make the topics being explored clearer to participants. For each of the given vignettes, we will ask participants what compensation and benefits they see as ethically appropriate and why.

**Suggestions on level of compensation and benefits on study participation**

How people would rate the burden, discomfort of different procedure or what they think about calculating compensation based on reimbursement, compensation and incentives?

What are the steps that should be followed when considering appropriate levels on compensation and benefits on study participation?

Would the necessary steps be the same if the study design is different?

THANK YOU

**Appendix 4: Topic guide for community representatives, community leaders and fieldworkers/nurses (chichewa)**

**KAFUKUFUKU WOFUNA KUPEZA MAGANIZO A ATHU AMENE AMAKHUDZIDWA PA AKAFUKUKU PA ZA MLINGO WOYENERERA WA ZIPEPESO KOMANSO PHINDU LIMENE ATENGA MBALI MU KAFUKUFUKU AMAPASIDWA ATALOWA MUKAFUKU.**

**woyimilira kafukufuku mudera, mafumu and Fieldworkers/nurses – zokambilana za mmagulu.**

**Kudziwa wa ntchito za kafukufuku mmadera**

**Ogwira tchito za kafukufuku mmadera:**

Ndi thawi yaitali bwanji immene mwakhala mukukhudzidwa/kugwira ntchito za akafukufuku mu bungwe limeneli la MLW?

Ndi akafukufuku ati amene mwakhudzidwa nawo?

Akafukufuku amenewa amatani?

*CAG (woimilira mmadera) komanso mafumu:*

Ndi akafukufuku ati amene mwakhudzidwa kapena amene achitika mmdera lanu/kapena amene mukawadziwa?

Mudaonapo akafukufuku akupereka zipepeso komanso phindu?

**MAGANIZO A ATHU AMENE AMAKHUDZIDWA PA AKAFUKUKU PA ZA MLINGO WOYENERERA WA ZIPEPESO KOMANSO PHINDU LIMENE OTENGA MBALI MU KAFUKUFUKU AMAPASIDWA ATALOWA MUKAFUKU**

Kodi akafukufuku amenewa amapereka zipepeso komanso phindu kwa atenga mbali?

Ngati zipepesokomanso phidu zinaperekedwa:

Ndi zipepeso kapena phindu la mtundu uti umene udaperekedwa?

Mumanva bwanji pamene zipepesozi komanso phindu zikamaperekedwa kwa atenga mbali?

Kodi chipepeso choyenerera ndi chiti, nanga ndichifukwa chiyani?

Mukuganiza kuti zikuyenera kuganiziridwa ndizitii pamene mulingo woyenerera wa chipepeso ukuganizilidwa?

Mukuganizila kuti mulingo wa chipepeso umatengera mtundu wa kafukufuku, kapena zimene muthu akuyenera kuchita mukafukufuku? Nanga ndichifukwa chiyani?

Kodi anthu ammadera mumawanva akunene kuti chiyani, molingana ndi zipepeso komanso phindu zimene zimaperekedwa kupyolera mu akafukufuku? zimasiyana bwanji?

**ZOKAMBILANA**

Zokambilanaidzaperekedwa kwa otenga mbali zo onetsera zinthunzi nthunzi zosiyana siyana za akafukufuku (kafukufuku wochitikira ku chipatala ndi kafukufuku wo chitikira mmadera) kunthandizira kuti mutu wakafukufukuyu ufikilidwe kwa atenga mbali.Pakhani iliyonse tidzawafunsa atembali za

zipepeso komanso phindu zimene zimene zimaperekedwa kwa atenga mbali kuti amaziona bwanji moyenerela nanga ndi chifukwa chiyani?

**MAGANIZOPA ZA MLINGO WOYENERERA WA ZIPEPESO KOMANSO PHINDU LIMENE ATENGA MBALI MU KAFUKUFUKU AMAPASIDWA ATALOWA MUKAFUKU.**

Ndi njira ziti zoyenerera zimene zikuyenera kutsatilidwa poganizila mulingo woyenerera pa zipepepeso komanso phindu limene atenga mbali mu kafukufuku amapasidwa atalowa mu kafukufuku. Kodi njira zoyenererazi zikuyenera kukhala zofanana ngakhale akafukufuku alindi zochita zosiyana siyana?

ZIKOMO

**Appendix 5: Topic guides for decliners and research participants**

**Research participants and people who decided not to take part in research - focus group guide**

**Views of research stakeholders about appropriate benefits and compensation for study participation**

**Experience of research**

What research projects have you been invited to take part in?

What was your decision about participation? What affected your decision?

Have you had any experiences of studies offering compensation or benefits?

**Views on levels of compensation and benefit on study participation**

Were these studies providing compensation or benefits to participants?

If compensation or benefits was provided:

What kind of compensation or benefit was shared?

How did you feel about providing these compensation or benefits to participants?

What do you think is fair compensation and why?

What do you think should be considered when deciding on compensation levels?

Do you feel compensation depends on study type or what participants have to do? If so how?

What reactions do you hear from other people regarding compensation and benefits offered by studies? How does this vary?

**Vignettes**

Vignettes will be presented to participants indicating scenarios with different types of research (e.g. clinical trial, community-based study, ill or healthy volunteers) to make the topics being explored clearer to participants. For each of the given vignettes, we will ask participants what compensation and benefits they see as ethically appropriate and why.

**Recommendations or suggestions on levels of compensation and benefits on study participation.**

How people would rate the burden, discomfort of different procedure or what they think about calculating compensation based on reimbursement, compensation and incentives?

What are the steps that should be followed when considering appropriate levels on compensation and benefits on study participation?

Would the necessary steps be the same if the study design is different?

THANK YOU.

**Appendix 6: Topic guides for decliners and research participants (Chichewa)**

**Zotsoglera zokambilana za mmagulu; ndi atenga mbali komanso amene adakana kutenga nawo mbali pa akafukufuku.**

**KAFUKUFUKU WOFUNA KUPEZA MAGANIZO A ATHU AMENE AMAKHUDZIDWA PA AKAFUKUKU PA ZA MLINGO WOYENERERA WA ZIPEPESO KOMANSO PHINDU LIMENE OTENGA MBALI MU KAFUKUFUKU AMAPASIDWA ATALOWA MUKAFUKU.**

**Kudziwa ntchito za kafukufuku mmadera**

Ndi akafukufuku ati amene mwakhudzidwa kapena amene achitika mmdera lanu/kapena amene mukawadziwa?

Maganizo anu anali otani pazakutenga kwanu mbali? Chidasitha maganizo anu ndi chiyani?

Mudaonapo akafukufuku akupereka zipepeso komanso phindu?

**MAGANIZO A ATHU AMENE AMAKHUDZIDWA PA AKAFUKUKU PA ZA MLINGO WOYENERERA WA ZIPEPESO KOMANSO PHINDU LIMENE OTENGA MBALI MU KAFUKUFUKU AMAPASIDWA ATALOWA MUKAFUKU**

Kodi akafukufuku amenewa amapereka zipepeso komanso phindu kwa atenga mbali?

Ngati zipepesokomanso phidu zinaperekedwa:

Ndi zipepeso kapena phindu la mtundu uti umene udaperekedwa?

Mumanva bwanji pamene zipepesozi komanso phindu zikamaperekedwa kwa atenga mbali?

Kodi chipepeso choyenerera ndi chiti, nanga ndichifukwa chiyani?

Mukuganiza kuti zikuyenera kuganiziridwa ndizitii pamene mulingo woyenerera wa chipepeso ukuganizilidwa?

Mukuganizila kuti mulingo wa chipepeso umatengera mtundu wa kafukufuku, kapena zimene muthu akuyenera kuchita mukafukufuku? Nanga ndichifukwa chiyani?

Kodi anthu ammadera mumawanva akunene kuti chiyani, molingana ndi zipepeso komanso phindu zimene zimaperekedwa kupyolera mu akafukufuku? zimasiyana bwanji?

**ZOKAMBILANA**

Zokambilanazidzaperekedwa kwa otenga mbali zo onetsera zinthunzi nthunzi zosiyana siyana za akafukufuku (kafukufuku wochitikira ku chipatala,okhudzana ndi odwala, kapenawogwira ntchito ya zaumoyo mozipereka ndi kafukufuku wo chitikira mmadera) kunthandizira kuti mutu wakafukufukuyu ufikilidwe kwa atenga mbali.Pakhani iliyonse tidzawafunsa atembali za zipepeso komanso phindu zimene zimene zimaperekedwa kwa atenga mbali kuti amaziona bwanji moyenerela nanga ndi chifukwa chiyani?

**MAGANIZOPA ZA MLINGO WOYENERERA WA ZIPEPESO KOMANSO PHINDU LIMENE OTENGA MBALI MU KAFUKUFUKU AMAPASIDWA ATALOWA MUKAFUKU.**

Ndi njira ziti zoyenerera zimene zikuyenera kutsatilidwa poganizila mulingo woyenerera pa zipepepeso komanso phindu limene atenga mbali mu kafukufuku amapasidwa atalowa mu kafukufuku. Kodi njira zoyenererazi zikuyenera kukhala zofanana ngakhale akafukufuku alindi zochita zosiyana siyana?

**Zikomo**
